# Improvement of perceived cochlear implant sound quality through individualized psychoacoustic-based frequency fitting

**DOI:** 10.1016/j.zemedi.2025.02.001

**Published:** 2025-02-27

**Authors:** Tobias Rader, Lisa Lippl, Joachim Müller

**Affiliations:** a Division of Audiology, Department of Otorhinolaryngology, LMU University Hospital, Ludwig-Maximilians-Universität München, Munich, Germany; b MED-EL Deutschland GmbH, Starnberg, Germany; c Department of Otorhinolaryngology, LMU University Hospital, Ludwig-Maximilians-Universität München, Munich, Germany

**Keywords:** Cochlear implant, Fitting procedure, Psychoacoustic, Pitch matching, Music based

## Abstract

**Objective:**

In recent years, there has been a trend toward more individualization in the fitting of cochlear implants (CI). Here, a new individualized approach to frequency band allocation was used. This approach is based on binaural perceptual pitch matching.

**Patient:**

The patient had congenital bilateral progressive sensorineural hearing loss due to Usher Syndrome. He had used hearing aids in both ears since the age of 4 years. In his mid-40s, he received a CI on his right ear and, ten months later, a second CI on the left ear.

**Intervention:**

Adjustments to the frequency band allocations were made, guided by the binaural perceptual pitch matching of piano notes. For the first CI, pitch matching was performed using the contralateral ear as the reference, which had preserved low-frequency residual hearing (bimodal pitch matching). For the second CI, pitch matching was performed using the first implanted side as the reference (bilateral electrical pitch matching).

**Results:**

The final frequency band allocation adjustments were always shifted toward lower frequencies relative to the default band allocations. The adjustments were larger in magnitude for the second CI compared to the first CI. Speech perception scores generally increased over the course of rehabilitation and were higher with the individualized fitting compared to the default fitting. The subjective sound quality was reportedly greatly improved with the individualized fitting.

**Conclusions:**

Individualized psychoacoustic frequency-based fitting can yield improvements in the perceived sound quality with a CI. However, this method requires significant residual hearing in at least one ear, and the patient must have relatively fine pitch discrimination abilities.

## Objective

As part of their sound coding strategies, cochlear implant (CI) audio processors separate the acoustic input signal into distinct frequency bands, each of which is then allocated to the corresponding electrode contact. All CIs are initially programmed with a default frequency allocation table which can be modified using the clinical fitting software.

Mismatches can occur between frequency band assigned to an electrode contact and the range of perceived frequencies elicited by that electrode contact. Mismatches can alter the perception of pitch, inhibit binaural fusion, and may also impact sound quality and speech perception [1], [2], [3], [4]. One source of these mismatches may be the use of the Greenwood function for place-pitch mapping, which can yield frequency allocations that are inappropriately high [5], [6], [7], [8].

A potential alternative to place-pitch mapping functions is the use of individualized psychoacoustic fitting. This can be accomplished when there is sufficient residual hearing in the contralateral ear for pitch matching studies. Here we report the results of such a music based fitting procedure.

## 2 Patient

The patient, a male in his mid-40s, presented with congenital bilateral profound sensorineural hearing loss due to Usher Syndrome. Hearing aid use began at the age of 4 years. Despite his hearing loss, this patient developed a musical talent and could play several instruments. He had been implanted on the right side with a MED-EL SYNCHRONY2 CI with a 31.5 mm FLEXSOFT array. The array was fully inserted, verified by X-ray imaging with Stenvers view [9].

In the contralateral left ear, the hearing thresholds were 15–30 dB HL between 125 and 1 kHz and ≥65 dB HL between 1.5 and 9 kHz. In this ear he had worn a hearing aid (PHONAK Naida B70 SP) for a duration of 1.5 years.

Due to the progressive nature of his hearing loss, and to the degree of low-frequency residual hearing in the contralateral ear, the patient was offered to transition to electric-acoustic stimulation in that ear. However, the patient opted instead for a conventional CI partly due to concerns over his progressive loss of both vision and hearing.

Ten months after the first CI, the patient received a second CI in the left ear; a SYNCHRONY2 with the FLEXSOFT array. Following this, speech comprehension and reported enjoyment of music rapidly developed beyond what had been achieved with the hearing aids. After the second surgery, no residual hearing remained in either ear. SONNET2 audio processors with FS4 speech coding strategies were used in both ears. The stimulation rates of the electrodes were not modified. The number of fine structure channels remained unchanged at four during all fitting sessions. The electrical thresholds (T-values) and MCL-values (most comfortable loudness) were checked during the adaptation sessions and adjusted slightly if necessary.

## 3 Intervention

Pitch-matching experiments were performed after the first implantation. The patient was asked to compare the perceived pitch of two piano notes, played sequentially by himself on two keyboards, as heard the first through the CI (using the FS4 coding strategy) and the second through the non-implanted ear. He changed the second note on the keyboard until it matched the pitch of the first note. The patient documented the fundamental frequencies of the notes played and the fundamental frequencies of the corresponding pitches he perceived with each listening modality. For example when the patient was presented with the piano note c3 (1047 Hz), he stated that the perceived pitch via the CI was perceived as a2 (880 Hz) about three semitones lower than via the non-implanted ear.

Using the clinical fitting software of the manufacturer, we adjusted the center frequencies of the filter bands for the CI to attempt to match the pitch perceived with the unimplanted ear. The upper and lower cutoff frequencies of each frequency band were also changed so that that no gaps occurred in the filter bank, and so the shift was approximately equal for neighboring channels. The overall frequency range was kept at the default 70–8500 Hz.

After the second implantation, pitch-matching experiments were again conducted, this time with the left ear, using the first-implanted right ear as the perceptual reference.

## 4 Main outcome measures

The main outcome measures were the upper, lower, and center frequency allocations of each electrode contact, as well as scores on the Freiburg monosyllabic speech perception test (FMT) [10] and the subjective feedback of the patient regarding the sound quality with his devices.

## 5 Results

For the right ear, frequency allocations were changed across three post-operative fitting sessions (denoted F-I to F-III). The first session was performed two months after surgery. The center frequencies of channels 4 and 5 were both reduced by 16 Hz. Three months after surgery (F-II), the center frequencies of channels 3 and 5–12 were also reduced by 4–860 Hz and the center frequency of channel 4 was increased by 8 Hz. Six months after surgery (F-III), the center frequencies of channels 3–5, 9, and 10 were reduced by 10–884 Hz.

**Figure 1:**
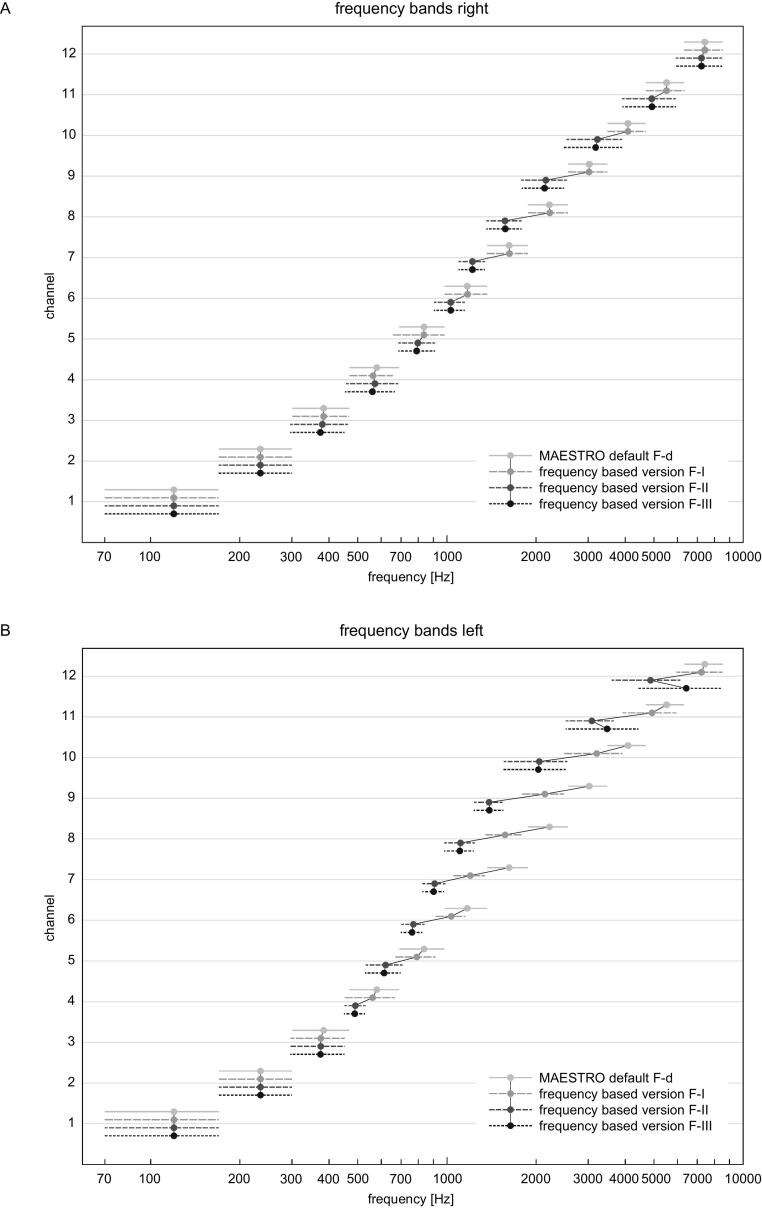
Frequency bands over the time for (A) right and (B) left side CIs. The grey line represents the default frequency bands of the fitting software (MAESTRO). The dashed and dotted lines show the frequency bands after individualized psychoacoustic fitting. Dots represent the center frequencies of each channel; the length of the line represents the length of each frequency band (upper and lower cutoff frequency). F-I, F-II, and F-III are the first, second, and third individualized fittings, respectively. The center frequencies of the different fittings are connected by lines.

Fig. 1A shows the original (default) and psychoacoustic-based frequency band allocations of the right CI over time. The frequency adjustments on the right ear ranged from 4 Hz to 884 Hz (0.02 to 0.5 octaves) lower than the corresponding default center frequencies. There were no frequency adjustments to channels 1 and 2. With the final individualized map, the greatest frequency shifts were made at channel 7 (406 Hz, 0.42 octaves), channel 8 (649 Hz, 0.5 octaves), and channel 9 (884 Hz, 0.5 octaves).

After the second CI implantation, individualized psychoacoustic frequency fitting was also performed on the left ear, using the implanted right ear as the reference. Two months after surgery (F-I), the center frequencies of channels 3–12 were reduced by 10–890 Hz. One month later (F-II), the center frequencies of channels 4-12 were further reduced by 90–2510 Hz. At six months after surgery (F-III), the frequency bands of channels 2, 3, 5–8, 10 were reduced by 2–2036 Hz and the center frequencies of channels 9, 11, and 12 were increased by 7–1584 Hz.

Fig. 1B shows the original (default) and psychoacoustic guided frequency band allocations of the left CI over time. The frequency adjustments on the right ear ranged from 2 to 2510 Hz (0.01 to 1.11 octaves) lower than the corresponding default center frequencies. There were no frequency adjustments to channel 1. The final individualized map had the greatest frequency shifts at channel 8 (1111 Hz, 1.0 octaves), channel 9 (1620 Hz, 1.11 octaves), and channel 10 (2036 Hz, 1.0 octaves).

**Figure 2:**
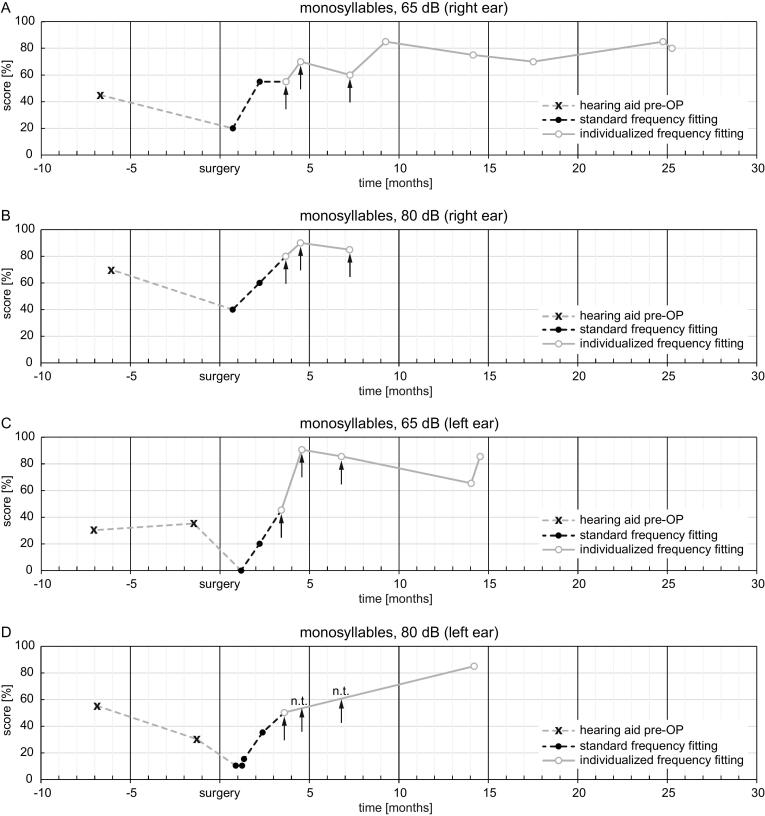
Freiburg monosyllable test scores in free field at (A) 65 dB, right ear; (B) 80 dB, right ear; (C) 65 dB, left ear; (D) 80 dB, left ear. Grey dashed lines show pre-operative aided scores. The black dashed lines show the scores with the default frequency allocations. The grey lines show the scores with the individualized frequency fittings. Arrows indicate the timepoints of F-I, F-II, and F-III for each ear. n.t.: not tested due to high scores at 65 dB.

Fig. 2 shows the aided speech perception scores on the FMT with each ear tested separately. Speech was presented in free field at 65 dB and at 80 dB. In general, FMT scores increased over time, with some variation which was attributed to acclimatization to the new fittings and to the measurement uncertainty of the FMT.

The results in speech perception scores do not indicate that the improvement in speech comprehension was caused by the individualized psychoacoustic-based frequency fitting. It is certainly overlaid by the rehabilitative learning process.

After these adjustments, the perceived sound quality of the CIs, especially with regard to playing the piano and to the enjoyment of music, was reported to be significantly better. According to the patient, the improvements which he perceived after adjustments to the first CI contributed to his decision to opt for a second CI on the contralateral ear.

## 6 Conclusions

This case illustrates the potential of individualizing fitting via psychoacoustic pitch matching. As reported in previous studies, the perceived pitch in this case was up to 1.11 octaves lower than predicted by the Greenwood function [6], [7], [8]. With both ears, all frequency shifts in the final maps were towards lower frequencies relative to their default allocations. The frequency shifts of the second CI were larger in magnitude compared to the first CI. The apical channels 1 and 2 provided the least perceptual mismatches with their default frequency allocations. These results are in accord with a previous pitch matching study [11], [12].

Individualized psychoacoustic fitting may provide a useful alternative to fitting methods that rely on pitch-place functions to assign frequencies to electrode contacts. In this case, the procedure yielded perceived improvements in both sound quality and the enjoyability of music.

Speech perception test scores generally increased over time, as scores were higher with the individualized fitting compared to with the default frequency allocation. However, it is not possible to determine what proportion of this improvement, if any, can be attributed to the individualized fitting and what proportion can be attributed to the general improvement of speech perception which is typically observed over the first few months of CI use.

This procedure has some limitations. These include the necessity for functional residual hearing in at least one ear. The listener must also be able to not only perceive pitch differences, but to accurately report their magnitudes, a skill typically reserved for trained musicians. The method is also rather time consuming and can be challenging for both the patient and audiologist. Nevertheless, this method can yield improvements in the perceived sound quality with the CI.

## CRediT authorship contribution statement

**Tobias Rader:** Writing – review & editing, Writing – original draft, Visualization, Validation, Supervision, Software, Resources, Project administration, Methodology, Investigation, Formal analysis, Data curation, Conceptualization. **Lisa Lippl:** Writing – review & editing, Writing – original draft, Validation, Methodology, Investigation, Formal analysis, Data curation. **Joachim Müller:** Writing – review & editing, Resources.

## Funding

This research received no external funding.

## Data Availability Statement

The anonymized patient data can be made available on request due to privacy/ethical restrictions.

## Declaration of competing interest

The authors declare the following financial interests/personal relationships which may be considered as potential competing interests: T.R. and J.M. received funding for other projects supported by different medical device companies. L.L. is an employee of MED-EL Deutschland GmbH, Starnberg, Germany.
